# Effects of Organic Selenium on the Physiological Response, Blood Metabolites, Redox Status, Semen Quality, and Fertility of Rabbit Bucks Kept Under Natural Heat Stress Conditions

**DOI:** 10.3389/fvets.2020.00290

**Published:** 2020-06-12

**Authors:** Nourhan S. Hosny, Nesrein M. Hashem, Amr S. Morsy, Zahraa R. Abo-elezz

**Affiliations:** ^1^Department of Animal and Fish Production, Faculty of Agriculture, Alexandria University, Alexandria, Egypt; ^2^Livestock Research Department, Arid Lands Cultivation Research Institute, City of Scientific Research and Technological Application (STR-City), Alexandria, Egypt

**Keywords:** heat stress, male, selenium, antioxidant, semen

## Abstract

Heat stress can impair the general health of rabbit bucks by disturbing physiological homeostasis with negative consequences in animal welfare and remarkable decline in reproductive performance. Selenium (Se) can control a number of vital biological processes. Thus, the effects of organic selenium (OSe) supplementation on the blood metabolites, redox status, semen quality, testicular histology, seminal plasma protein profile, and fertility of rabbit bucks kept under natural heat stress conditions were studied. Adult V-line male rabbits were fed a basal diet supplemented with 0.3 mg OSe/kg dry matter (DM) diet (OSe, *n* = 9) or not (control, CON, *n* = 9) for 12 weeks. The results showed that rabbits fed the OSe diet had 73.68 and 68.75% higher (*P* < 0.05) OSe concentrations in the blood serum and seminal plasma, respectively, than rabbits fed the CON diet. The OSe diet significantly decreased the rectal temperature and respiration rate and significantly increased the blood serum concentrations of total protein, albumin, glucose, and glutathione peroxidase compared to the CON diet. Rabbits fed the OSe diet had lower reaction times (12.53 vs. 5.84 s, ± 0.79, *P* < 0.01) and higher total functional sperm counts (116.74 vs. 335.23 × 10^6^/ml, ± 24.68*, P* < 0.001) and percentages of integrated sperm membranes (60.38 vs. 79.19%, ± 1.69, *P* < 0.01) than rabbits fed the CON diet. Rabbits fed the OSe diet had higher (*P* < 0.01) contents of seminal plasma total protein, albumin, alanine transaminase, fructose, and total antioxidant capacity and lower (*P* < 0.001) malondialdehyde (MDA) levels than those fed the CON diet. Rabbits fed the OSe diet had sperm cells with higher levels of integrated DNA than those fed the CON diet. The seminal plasma of rabbits fed the OSe diet contained four new proteins, with molecular weights of 19.0, 21.5, 30.0, and 44.0 kDa. The kindling rates, litter size, and weight at birth of females mated with males fed the OSe diet were significantly higher than those of females mated with males fed the CON diet. In summary, the inclusion of 0.3 mg OSe/kg DM diet of naturally heat-stressed rabbit bucks countered the negative impacts of elevated environmental temperature on physiological homeostasis, semen quality, and fertility.

## Introduction

Increasing global warming represents a real challenge to the livestock industry, particularly in arid and semi-arid regions. In rabbit farms, continuation of the breeding season could be restricted for several months due to heat stress (high ambient temperatures), which has negative effects on male fertility, resulting in pronounced economic loss (1). Heat stress alters testicular structure and weight, decreases sperm output, reduces sperm quality, and increases sperm morphological abnormalities and DNA fragmentation in male germ cells (2). The negative effects of heat stress on male fertility are partially related to the generation of reactive oxygen species (3). Accordingly, utilization of antioxidants might be a method to address the negative effects of heat stress on male fertility. Selenium (Se), in addition to its specific role in the maintenance of male fertility, is a powerful biological antioxidant mineral. The function of Se in the male reproductive tract is independent of its other physiological processes in the body; Se acts at the reproductive tissue level and is involved in both testosterone biosynthesis and the formation and development of spermatozoa (4). Moreover, Se is an integral component of at least 25 selenoproteins, including glutathione peroxidase (GPx) and other functional and structural proteins in the testis, epididymis, and sperm (5, 6). For example, GPX1 and GPX3 are expressed and located in the epididymal epithelia and sperm to protect the epididymal parenchyma and maturing sperm from oxidative stress. Also, GPX4 can protect the developing sperm from oxidative stress–induced DNA damage; it is also a structural component of the mitochondrial sheath in the sperm midpiece, consequently being an essential component for sperm stability and motility (7).

Dietary Se supplements are available in two forms: inorganic mineral salts (sodium selenite or selenate) and organic forms (selenomethionine from Se-enriched yeast). Several studies have reported that Se from organic sources is metabolized much more efficiently than the inorganic forms and could be efficiently utilized for synthesis of selenoproteins in conditions of stress (5, 7). There have been few studies, however, evaluating the effects of dietary Se on male rabbits' reproductive performance, and the results from these studies have been inconclusive. Abdulrashid and Juniper (2) reported that a supplemental dietary organic selenium (OSe) (selenomethionine) concentration at 0.3 mg/kg dry matter (DM) diet added to a control diet containing 0.4 mg Se/kg DM did not influence semen quality or reproductive performance of adult male rabbits under tropical conditions. Similarly, a supplemental dietary Se (sodium selenite, inorganic) concentration at 0.5 mg Se/kg DM diet added to a control diet containing 0.1 mg Se/kg DM diet did not improve the oxidative stability of cryopreserved sperm cells in rabbit bucks (8). On the other hand, Kamel (9) and Ewuolaand Akinyemi (10) found that supplemental dietary OSe (selenomethionine) at 0.6 mg/kg DM diet or at 0.4 mg/kg DM diet respectively significantly improved the semen quality and reproductive performance of male rabbits. In this study, we hypothesized that dietary Se supplementation in organic form may alleviate the heat stress–related decline in semen quality and fertility in rabbit bucks. Thus, the effects of OSe supplementation for 12 consecutive weeks (84 days, covering the duration of the spermatogenesis process of rabbits, about 60 days) on the blood metabolites, semen quality, seminal plasma protein profile, testicular histology, and fertility of rabbit bucks kept under natural heat stress conditions were evaluated.

## Materials and Methods

### Animal Husbandry and Experimental Design

**Figure 1:**
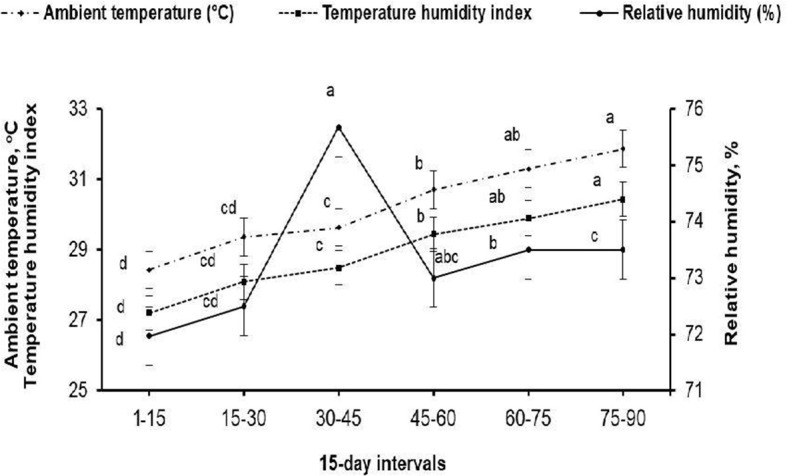
Mean ambient temperature (°C), relative humidity (%), and temperature–humidity index throughout the 15-days intervals of the experimental period. ^**(a−d)**^ Means (± pooled *SE*) of each variable marked with different superscript letters are significantly different (*P* < 0.001).

The present study was carried out at the Laboratory of Rabbit Physiology Research, Agricultural Experimental Station, Faculty of Agriculture, Alexandria University, Alexandria, Egypt. Rabbit bucks, and does subjected to a fertility test, used in this study were of V-line breeding, a maternal line selected based on litter size at weaning [Department of Animal Science, UPV, Valencia, Spain; (11)]. Eighteen sexually mature (7.5 ± 0.37 months old) rabbit bucks weighting 2.60 ± 0.10 kg at allocation (initial weight) were housed in the rabbitry under similar management and hygiene conditions. The maximum and minimum values of ambient temperature and relative humidity in the rabbitry were recorded daily during the experimental period (May to August) using an automatic computerized system for hydrothermography (Data logger-Log 100/110, Germany). The temperature–humidity index (THI) was calculated using the following equation: THI = db°C–[(0.31–0.31(RH))] × (db°C−14.4)], where db°C is the dry bulb temperature and RH is the relative humidity percentage/100. The calculated THI values were subsequently classified as follows: <27.8 = absence of heat stress; 27.8–28.9 = moderate heat stress; 28.9–30.0 = severe heat stress; and >30.0 = extremely severe heat stress (12). The means of ambient temperature (ranging from 28.93 to 31.30°C), relative humidity (ranging from 71.97 to 73.50%), and THI (ranging from 27.89 to 30.43) throughout the experimental period are shown in Figure 1. The mean values of THI obtained in this study were classified as moderate heat stress to extremely severe heat stress.

The rabbits were kept in individual galvanized wire cages (40 × 50 × 35 cm^3^). The cages were equipped with feeders and automatic drinkers that provided fresh tap water (*ad-libitum)*. Before allocation of rabbits into their respective experimental groups, libido, semen volume, sperm concentration, and sperm progressive motility were evaluated for each male and considered at allocation. The means (± pooled SEs; *P* > 0.05) of libido (± 0.56), semen volume (± 0.17), sperm concentration (± 21.17), and sperm progressive motility (± 2.74) were: 12.0 s, 0.63 ml, 454.5 × 10^6^/ml, and 66.73% for rabbits fed the CON diet; and 11.76 s, 0.59 ml, 470.3 × 10^6^/ml, and 69.0% for rabbits fed the OSe diet. The rabbits were equally divided into two groups and were fed either a control diet (control, CON, *n* = 9) or the CON diet supplemented with 0.3 mg OSe/kg DM diet (OSe, *n* = 9) for 12 consecutive weeks for data collection and for an additional week for performing fertility tests (week 13), during which the first 2 weeks were considered as an adaptation period. Se supplementation was performed in the form of Se-enriched yeast derived from a specific strain of *Saccharomyces cerevisiae*, CNCM I-3060 (Biosel Plus, Vetagri Consulting Inc., Brampton, Canada), loaded on wheat bran. The supplementation was added to the OSe diet during pellet diet formulation by replacing 5 g/kg of wheat bran with the same weight of Se-enriched yeast product (source of OSe). The CON diet was formulated to meet the nutritional requirements of rabbit bucks according to the National Research Council (13). The ingredients, analyzed chemical composition, and calculated metabolizable energy of the CON diet are shown in Table 1. The Se content in the CON diet was 0.034 mg/kg DM [determined following the method described by the Association of Official Analytical Chemists, (14)].

**Table 1:** Ingredients (g/kg), analyzed chemical composition [g/kg dry matter (DM)], and calculated metabolizable energy (MJ/kg DM) of the control diet.

**Item**	**Control diet**
**Ingredients (g/kg)**	
Barley	180
Wheat bran^a^	250
Yellow corn	60
Soy bean	180
Alfalfa hay	280
Molasses	30
Di-calcium phosphate	10
Sodium chloride	7
Vitamin–trace mineral premix mixture^b^	3
**Chemical Composition (g/kg DM)**	
Organic matter^c^	862
Ether extract^c^	21.0
Crude protein	182
Neutral detergent fiber^d^	301
Acid detergent fibrer^d^	166
Selenium (Se, mg/kg DM)^c^	0.034
Metabolizable energy (MJ/kg DM)^e^	11.29

a *To formulate an organic selenium (OSe) supplemented pellet diet, 5 g/kg of wheat bran was replaced with the same weight of Se-enriched yeast product*.

b *Each 3 kg of vitamin–trace mineral premix mixture contained the following: vitamin A 12,000,000 IU; vitamin D_3_ 2,000,000 IU; vitamin E 10,000 mg; vitamin K_3_ 2,000 mg; vitamin B_1_ 1,000 mg; vitamin B_2_ 5,000 mg; vitamin B_6_ 1,500 mg; vitamin B_12_ 10 mg; biotin 50 mg; choline chloride 250,000 mg; pantothenic acid 10,000 mg; nicotinic acid 30,000 mg; folic acid 1,000 mg; iron 30,000 mg; copper 10,000 mg; manganese 60,000 mg; iodine 1,000 mg; selenium 25 mg; cobalt 100 mg; zinc 50,000 mg*.

c *Analyzed according to the Association of Official Analytical Chemists (14)*.

d *Analyzed according to Van Soest et al. (15)*.

e *Calculated according to Nutrient Requirements of Rabbits (13)*.

### Selenium Concentration in Blood Serum and in Seminal Plasma

Blood serum and seminal plasma samples collected on weeks 0, 4, 8, and 12 were analyzed for Se concentrations using inductively coupled plasma-optical emission spectroscopy (ICP-OES, model prodigy-SPES, Berlin, Germany). The limit of detection for Se concentration calculated as a mean of duplicate samples of three runs was 0.004 ppm/ml (coefficient of variation 0.01–0.02%). The serum and seminal plasma samples (2 ml) were digested using a microwave digester with 10 ml of concentrated nitric acid and 2 ml of hydrogen peroxide (30%), and then the mixture was cooled at room temperature (16).

### Physiological Variables and Blood Serum Biochemical Attributes

Rabbits were weighed weekly in the morning before being offered the diet. The feed intake was calculated weekly by subtracting the unconsumed feed from the total amount offered during this period and recorded as g/day. Rectal temperature (°C) and respiration rate (breaths/min) were recorded weekly in the morning. Rectal temperature of each rabbit was measured by using a clinical digital thermometer that was gently pushed and attached to rectal wall until a fixed reading was obtained. Respiration rate was determined by counting the number of flank movements throughout 1 min.

Blood samples were collected bi-weekly (on weeks 2, 4, 6, 8, 10, and 12) from the ear vein of each rabbit into non-heparinized tubes during the entire experimental period. Blood samples were centrifuged at 700 × g for 20 min at 4°C. The separated serum was collected and stored at −20°C until subsequent analyses. The blood serum was colorimetrically analyzed for total protein content, albumin content, glucose content, and glutathione peroxidase or GPx (GSH-px) activity using commercial kits obtained from Bio-diagnostic (Giza, Egypt). The linearity of the methods was up to 10.0 g/dl, 7.0 g/dl, and 500 mg/dl for total protein content, albumin content, and glucose content, respectively. The activity of GSH-px was indirectly determined by recording the rate of reduction of oxidized glutathione by GSH-px yielding oxidized NADP. This reaction is accompanied by a decrease in absorbance at 340 nm, providing a spectrophotometric means for monitoring GSH-px enzyme activity. The molar extinction coefficient for NADPH is 6,220 M/cm at 340 nm.

The blood serum testosterone concentration was measured using a solid-phase enzyme immunoassay test kit (BioCheck, Inc., Foster City, CA). The minimum detectable concentration of the assay was 0.05 ng/ml. The intra- and inter-assay coefficients of variation were 7.43 and 5.23%, respectively.

### Libido, Semen Physical Evaluation, and Sperm DNA Fragmentation

Before starting the treatment, the bucks were trained to serve an artificial vagina. Libido (sexual desire) was measured using a stopwatch and expressed as reaction time (the time in seconds from the insertion of the female into the male cage to ejaculation by the male) (1). Semen ejaculates were collected weekly (from week 2 to 12) using an artificial vagina maintained at 45 to 46°C using a teaser doe. The semen ejaculate volume was determined after removal of gel mass (if present) by a graduated tube that was directly connected to the artificial vagina. A trained technician was devoted to assessing sperm concentration, progressive motility, viability, and morphology following the guidelines of International Rabbit Reproduction Group (17). Sperm concentration was determined by using an improved Neubauer hemocytometer slide (GmbH + Co., Hamburg, Germany) after dilution (1:100) in formaldehyde phosphate buffered saline solution. Sperm motility (progressive motility) was immediately assessed after semen collection in several microscopic fields by visual examination at 100 × magnification using a 37°C warm-plate-equipped light microscope (GLCD-120, GIPPON INC., Japan) with classifications of subjective assessments ranging from 0 to 100%. Approximately 200 sperm cells per sample were examined with a light microscope (100 × magnification) for morphology (normal morphology, head abnormality, and tail abnormality), viability (live: non-stained sperm cells, or dead: purple stained sperm cells), and acrosome integrity (intact acrosome: sperm cells with acrosome cap, or non-intact acrosome: sperm cells without acrosome cap) after staining with an eosin-nigrosine blue dye mixture. The hypo-osmotic swelling test (HOST), indicating functional membrane integrity, was performed on the semen samples by incubating 30 μl of semen with 300 μl of hypo-osmotic solution (9 g of fructose plus 4.9 g of sodium citrate/distilled water; 100 mOsm/kg) at 37°C for 1 h (3). For a complete overview of the quality of the semen ejaculate, the total functional sperm fraction (TFSF) was calculated as follows: TFSF = total sperm output (10^6^/ejaculate) × percentage of progressive motility × percentage of normal sperm morphology (3). For the sperm DNA fragmentation test, sperm cells were harvested by centrifugation at 700 × g for 20 min. The sperm cells collected during the experimental period from each buck were pooled and used for DNA extraction (QIAamp DNA Mini Kit, Cat. No. ID: 51304, Hilden Germany). The DNA samples were mixed with 6 × loading dye buffer (Promega, USA). The loaded gels were run in an electrophoresis unit (Invitrogen Novex Mini-Cell, USA) at room temperature in constant voltage mode at 140 V for 1 h. The molecular weight of each separated band was identified using DNA marker ladder standards ranging from 100 to 1,500 bp (Cambridge Reagents LTD, UK). The isolated DNA bands were photographed using a gel documentation system (Alpha-Chem Imager, USA). The density of each separated DNA fragment was determined using TOTALLAB software (Principal Experimental Officer, Molecular and Clinical Pharmacology, Institute of Translational Medicine, University of Liverpool, UK).

### Seminal Plasma Analyses

Seminal plasma was obtained by centrifugation of semen samples at 700 × g for 20 min. The samples were stored at −20°C, pending analysis. Seminal plasma samples were colorimetrically analyzed bi-weekly (on weeks 2, 4, 6, 8, 10, and 12) for determination of total protein content, albumin content, alanine transaminase (ALT) content, fructose content, total antioxidant capacity (TAC), and malondialdehyde (MDA) content using a commercial kit (Bio-diagnostics, Giza, Egypt) and instructions of the manufacturers provided in the kits. The linearity of the methods was up to 120 U/ml, 1,000 mg/dl, 2 mM/l, and 100 nmol/ml for ALT, fructose content, TAC and MDA, respectively.

The seminal plasma samples collected during the experimental period from each rabbit were pooled to obtain enough quantity for generating a seminal plasma protein profile using SDS-PAGE (18). The proteins from the seminal plasma were separated by adding lysis buffer (38.8 mM NH_4_Cl, 2.5 mM KHCO_3_, and 1 mM EDTA; pH = 8) into the seminal plasma samples, after which the mixture was incubated at room temperature and then centrifuged at 12,000 × g for 5 min at 4°C. The seminal plasma proteins were then mixed with the sample dye (50 mM Tris-HCl, 1 mM MB-mercapto, 0.1% bromo-phenol blue, 2% SDS, and 10% glycerol) at a 4:1 ratio and heated in a water bath at 95°C for 10 min. Then, the plasma protein samples and protein markers were electrophoresed using polyacrylamide gels (30% w/v resolving gel and 5% w/v stacking gel). The loaded gels were run in an electrophoresis unit (Invitrogen Novex Mini-Cell, USA) at room temperature in constant voltage mode at 70 V until the dye front entered the resolving gel; thereafter, electrophoresis was carried out at 100 V for 2 h. After electrophoresis, the gels were stained with Coomassie brilliant blue (Sigma, St. Louis, MO, USA) overnight and then distained with three changes of distilled water at room temperature. The molecular weight of each separated protein was identified using a multicolor broad-range protein marker (11–245 kDa; Pre-stained Protein Marker, Tiangen Biotech Co. Ltd., Beijing, China). Densitometric analysis of the separated seminal plasma proteins was carried out using TOTALLAB software (Principal Experimental Officer, Molecular and Clinical Pharmacology, Institute of Translational Medicine, University of Liverpool, UK). The relative protein fraction (%) of each separated protein was estimated by dividing the protein intensity signal by the total intensity signal of the lane (the summation of the peak intensity of all bands detected in the lane).

### Fertility Evaluation of Bucks

After the end of the last semen ejaculate evaluation, additional semen samples were collected at week 13 and pooled from each experimental group to evaluate the fertility of the rabbit bucks, following guidelines of the International Rabbit Reproduction Group (17). Insemination doses of 0.2 ml containing 15 × 10^6^ sperm cells were prepared. Forty-six nulliparous sexually mature (6.32 ± 0.41 months old) female rabbits weighting 2.86 ± 0.17 kg were subjected to estrous synchronization by treating each female with equine chorionic gonadotropin (eCG; 25 IU im; Gonaser®, Hipra, Spain); 48 h later, gonadotropin-releasing hormone (GnRH; 0.8 μg of buserelinim; Receptal, Boxmeer, Holland) was administered to induce ovulation, and females were immediately artificially inseminated (19). Pregnancy was diagnosed on day 10 post-insemination by abdominal palpation. The number of does who delivered and numbers of total litters, live litters, and dead litters at birth as well as litter weight at birth were recorded. Previous results were used to evaluate the fertility of does in each group as follows: conception rate, % = no. of pregnant females on day 10/total no. of inseminated females × 100; kindling rate, % = no. of delivered females/total no. of pregnant females × 100; abortion rate, % = no. of females aborted/total no. of pregnant females × 100; litter size at birth = total no. of litters born/total no. of kindling females; and no. of dead litters at birth = no. of dead litters at birth/total no. of litters at birth.

### Testicular Histomorphological Evaluation

At the end of the experiment, three rabbits randomly chosen from each group were euthanized. The testes of each buck were dissected and stored in 10% (w/v) formalin solution. Testicular histological sections were prepared using the paraffin block technique (20). Histomorphological evaluation of the testicular sections was carried out by a highly skilled technician with the aid of a light microscope (Olympus BX53, Tokyo, Japan) fitted with a digital camera (Olympus-DP80, Tokyo, Japan) to capture representative images. The histomorphological evaluation of the testicular sections was performed by examining 25 fields in five histological sections for each buck. The morphology of somniferous tubules and different germ cells within the somniferous tubules was used as an indicator to the competence of seminiferous tubules and spermatogenesis process. A score was given to describe the observed differences between the testicular sections of the experimental groups (++, normal, and ++++, increased).

### Statistical Analysis

Before statistical analysis, the percentages of motile, live, and abnormal sperm cells and the percentage of sperm with intact acrosomes and integrated membranes were subjected to square root transformation to approximate a normal distribution. The data, including the blood serum and seminal plasma Se contents, biochemical levels, and semen characteristics, were analyzed using the MIXED procedure for repeated measurements (21). In the statistical model, treatment (CON vs. OSe), week of sampling (12 weeks for semen physical variables and six for blood serum and seminal plasma biochemical levels), and the interaction between the two were introduced as fixed effects, while rabbit individual was introduced as a random effect. The equation of the MIXED procedure was: y_ijk_ = μ + T_i_ + W_j_ + (TW)_ij_ + e_ijk_, in which y_ijk_ is the observed value of the dependent variable, μ is the overall mean, T_i_ is the fixed effect of the ith treatment, W_j_ is the fixed effect of the jth week (time) of data collection, (T × W)_ij_ is the interaction between treatment and week (time) of data collection, and e_ijk_ is the residual error. The fixed effects of treatment (CON vs. OSe) on sperm DNA damage, seminal plasma protein fraction, litter size, number of dead litters, and litter weight at birth were tested by the generalized linear, GLM, procedure using the following model: y_ij_ = μ + T_i_ + e_ij_, in which y_ij_ is the observed value of the dependent variable, μ is the overall mean, T_i_ is the fixed effect of the ith treatment, and e_ij_ is the residual error (21). Conception, kindling, and abortion rates were analyzed using the Chi-square test (PROC FREQ). Differences among treatment group means were tested by Duncan's new multiple range test (21). All the results were expressed as the least square mean (LSM) ± pooled standard error (SE). Statistical significance was accepted at *P* < 0.05.

## Results

### Effects on Selenium Concentration in Blood Serum and in Seminal Plasma

**Figure 2:**
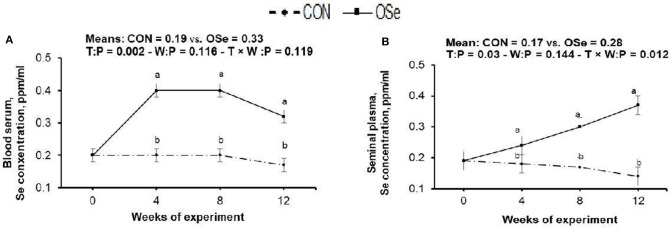
Effects of treatment (T: control, CON, vs. organic selenium, OSe) and T × week (W) interaction on blood serum selenium content (A) and on seminal plasma selenium content (B) in rabbit bucks. The results are least square means ± pooled *SE, n* = 9/treatment/time point. ^**(a,b)**^ Means within the same week of the experimental period with different superscript letters differ at *P* < 0.05.

The effects of OSe supplementation on the Se concentrations in blood serum and in seminal plasma are shown in Figure 2. Rabbits fed the OSe diet had 73.68 and 68.75% (*P* < 0.05) higher OSe concentrations in the blood serum and seminal plasma, respectively, than rabbits fed the CON diet. These increases were observed from weeks 4 to 12 of the experimental period.

### Effects on Physiological and Blood Biochemical Variables

**Figure 3:**
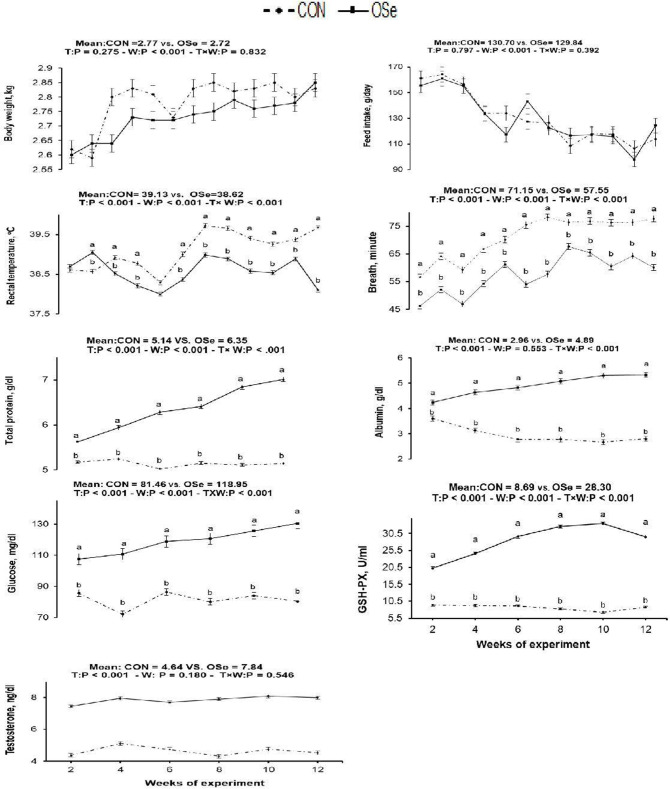
Effects of T (CON vs. OSe) and T × week (W) interaction on body weight, feed intake, respiration rate, rectal temperature, and the blood biochemical variables, including total protein content, albumin content, glucose content, glutathione peroxidase (GSH-PX) activity, and testosterone content in rabbit bucks. The results are least square means ± pooled *SE, n* = 9/treatment/time point. ^**(a,b)**^ Means within the same week of the experimental period with different superscript letters differ at *P* < 0.05.

The effects of OSe supplementation on the physiological variables and blood serum metabolites are shown in Figure 3, respectively. There was no significant difference in body weight or feed intake between the two experimental groups. However, OSe supplementation decreased (*P* < 0.001) the rectal temperature and respiration rate. The blood serum total protein content, albumin content, GSH-Px activity, glucose content, and testosterone content determined at weeks 2, 4, 6, 8, 10, and 12 were (*P* < 0.001) higher in the rabbits fed the OSe diet than in those fed the CON diet.

### Effects on Libido, Semen Properties, and Fertility

**Figure 4:**
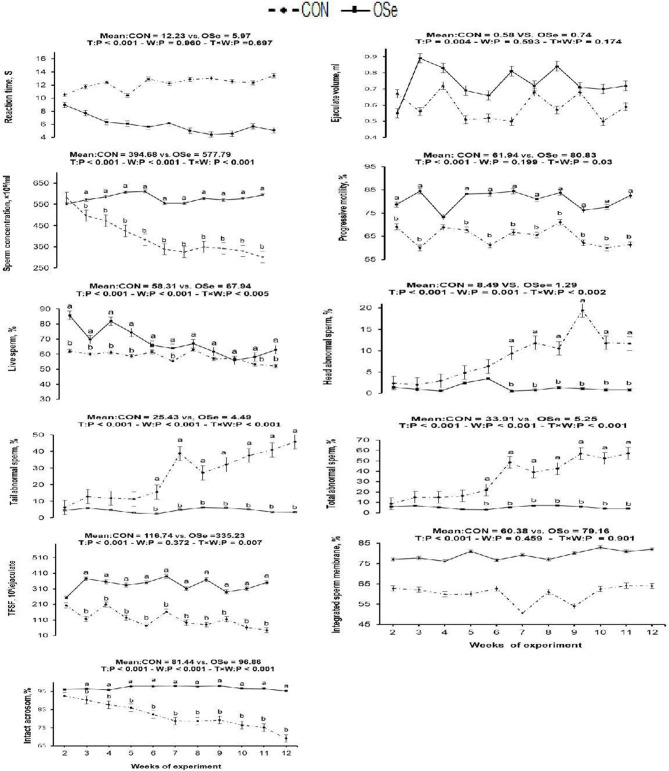
Effects of T (CON vs. OSe) and T × week (W) interaction on reaction time, ejaculate volume, progressive motility, and sperm concentration and percentages of live sperm, head abnormal sperm, tail abnormal sperm, total abnormal sperm, total functional sperm fraction (TFSF), integrated sperm membrane, and intact acrosome in rabbit bucks. The results are least square means ± pooled *SE, n* = 9/treatment/time point. ^**(a,b)**^ Means within the same week of the experimental period with different superscript letters differ at *P* < 0.05.

**Figure 5:**
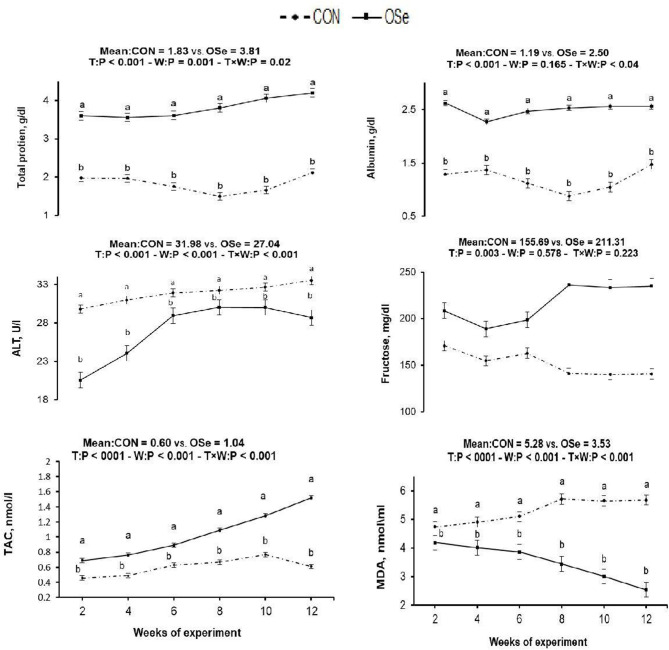
Effects of treatment (T: CON vs. OSe) and T × week (W) interaction on seminal plasma total protein content, albumin content, alanine transaminase (ALT) content, fructose content, total antioxidant capacity (TAC), and malondialdehyde (MDA) content in rabbit bucks. The results are least square means ± pooled *SE, n* = 9/treatment/time point. ^**(a,b)**^ Means within the same week of the experimental period with different superscript letters differ at *P* < 0.05.

**Figure 6:**
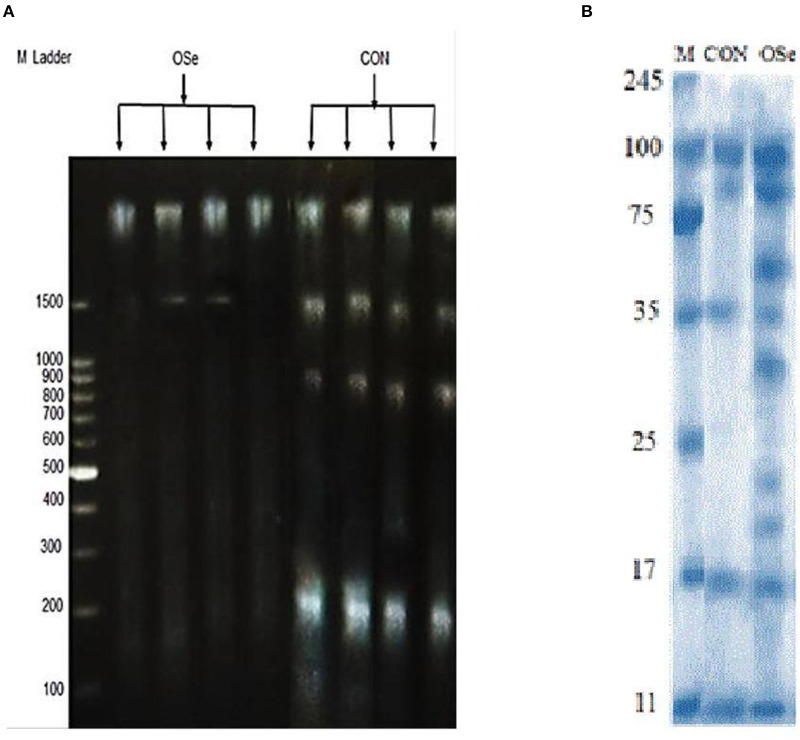
DNA degradation test **(A)** of sperm cells and SDS-PAGE **(B)** of seminal plasma proteins collected from rabbits fed the CON diet and rabbits fed the OSe diet. M Ladder represents DNA molecular weight markers.

The effects of dietary OSe supplementation on the libido and semen physical variables of rabbits are shown in Figure 4. Throughout the experimental period, rabbits fed the OSe diet had lower (*P* < 0.05) reaction times (better libido) and higher ejaculate volumes (*P* = 0.004) than those fed the CON diet. During the experimental period, the OSe diet improved (*P* < 0.001) the sperm concentration and percentages of sperm progressive motility, live sperm, sperm with integrated membranes, and total functional sperm compared to the values obtained with the CON diet. Significant reductions in the percentages of abnormal head sperm (*P* = 0.002) and abnormal tail sperm (*P* < 0.001) were observed for rabbits fed the OSe diet from weeks 5 to 12 of the experimental period compared to the values for rabbits fed the CON diet. From week 2, the percentage of sperm with intact acrosomes increased (*P* < 0.001) in the rabbits fed the OSe diet. Rabbits fed the OSe diet had higher (*P* < 0.01) seminal plasma total protein content, albumin content, fructose content, and TAC and lower ALT and MDA contents (*P* < 0.001) than those fed the CON diet (Figure 5). The DNA degradation test (Table 2; Figure 6A) showed that rabbits fed the OSe diet had sperm cells that yielded higher density (65.42) for the high-molecular-weight DNA (> 1,500 bp, integrated DNA) than those fed the CON diet (52.09), whereas rabbits fed the CON diet had sperm cells that yielded high density for DNA fragments at 1,500, 900, and 200 bp. SDS-PAGE analysis of the seminal plasma proteins (Table 2; Figure 6B) showed that the seminal plasma of OSe-treated rabbits contained four new proteins, at molecular weights of 19.0, 21.5, 30.0, and 44.0 kDa. On the other hand, the seminal plasma of CON-treated rabbits contained higher (*P* < 0.05) relative areas for two proteins at molecular weights of 16.0 and 35.0 kDa. The conception rate did not differ between the two experimental groups. Females mated with rabbits fed the OSe diet had significantly higher kindling rates, litter sizes, and litter weights at birth and lower abortion rates than those mated with rabbits fed the CON diet (Table 3).

**Table 2:** Sperm DNA fragmentation and seminal plasma protein profile of semen collected from rabbits fed the control (CON) diet and rabbits fed the organic selenium (OSe) diet.

**Variable**	**Treatment (*****n*** **=** **9/group)**		**Pooled *SE*^1^**	***P*-value**
	**CON**	**OSe**		
**Sperm DNA fraction**	**Optical density**			
Intact DNA	52.09^b^	65.42^a^	0.14	<0.001
1,500 bp	53.49^a^	10.66^b^	1.15	<0.001
900 bp	50.69^a^	0.00^b^	1.04	<0.001
200 bp	67.91^a^	0.00^b^	0.62	<0.001
**Protein molecular weight**	**Fraction, %**			
94 kDa	19.68	19.92	0.48	0.975
76.5 kDa	15.13	15.47	0.59	0.963
44 kDa	0.00^b^	12.51^a^	1.19	0.002
35 kDa	32.86^a^	2.85^b^	1.28	0.052
30 kDa	0.00^b^	8.81^a^	1.09	<0.001
21.5 kDa	0.00^b^	10.65^a^	1.14	0.001
19 kDa	0.00^b^	4.89^a^	2.12	0.020
16 kDa	19.93	11.68	0.40	0.086
11 kDa	12.40	13.21	0.49	0.870

1 *Pooled SE = pooled standard error*.

a,b *Means within row marked with different superscript letters are significantly different at P <0.05*.

**Table 3:** Conception rate, kindling rate, abortion rate, and least square means for litter size, number of dead litters, and litter weight for females mated with rabbits fed the control (CON) diet and the organic selenium (OSe) diet.

**Variable^1^**	**Treatment**		**Pooled *SE*^2^**	***P*-value**
	**CON (*n* = 23)**	**OSe (*n* = 23)**		
Conception rate, %	43.5 (10/23)	56.5 (13/23)	–	0.266
Kindling rate, %	60.0^b^ (6/10)	100^a^ (13/13)	–	0.025
Abortion rate, %	40.0^a^ (4/10)	0.0^b^ (0/13)	–	0.023
Litter size at birth, %	3.75^b^	6.15^a^	0.01	0.038
No. of dead litters at birth	0.88	1.31	0.27	0.610
Litter weight at birth, g	137^b^	279^a^	0.18	0.002

1 *Conception rate, % = no. of pregnant females on day 10/total no. of inseminated females × 100; kindling rate, % = no. of delivered females/total no. of pregnant females × 100; abortion rate, % = no. of females aborted/total no. of pregnant females × 100; litter size at birth = total no. of litters born/total no. of kindling females; and no. of dead litters at birth = no. of dead litters at birth/total no. of litters at birth*.

2 *Pooled SE = pooled standard error*.

a,b *Means within row marked with different superscript letters are significantly different at P <0.05*.

### Effects on Testicular Histology

**Figure 7:**
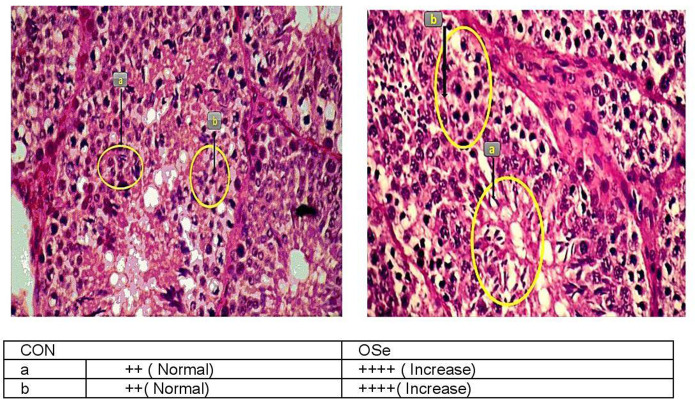
Histological evaluation of testicular cross section of rabbits fed the CON diet and rabbits fed the OSe diet. Mayer's hematoxylin and eosin, ×200. Sperm **(a)** and spermatid **(b)**.

The histological evaluation of the testicular cross sections is shown in Figure 7. In both treatments, testes showed normal histological appearance. However, seminiferous tubules of rabbits fed the OSe diet were filled and crowded by spermatid and sperm cells compared to those fed the CON diet.

## Discussion

Se is one of the most important trace elements and is required by mammals because of its involvement in many pivotal physiological processes, such as reproduction, immunity, and growth (7). Previous studies have shown that the basal Se concentration in the rabbit diet is between 0.01 and 0.15 mg/kg DM diet (22, 23), with an advisable concentration at 0.05 mg/kg DM diet for growing rabbits (24) or 0.08 mg/kg DM diet for lactating does (13). However, none of these studies have recommended a definite basal Se concentration in the diets of adult male rabbits; also, controversial findings related to the optimum dose of dietary Se and the importance of Se supplementation for the health and fertility of adult male rabbits have been obtained. Abdulrashid and Juniper (2) reported that supplemental dietary OSe (selenomethionine) concentration at 0.3 mg/kg DM diet added to a diet containing 0.4 mg Se/kg did not induce any enhancements in semen quality or reproductive performance of adult male rabbits under tropical conditions. On the other hand, Kamel (9) and Ewuolaand Akinyemi (10) found that supplemental dietary OSe (selenomethionine) at 0.6 mg/kg DM diet or at 0.4 mg/kg DM diet, respectively (basal diet Se content was not shown in both studies), significantly improved semen quality and reproductive performance of male rabbits. The contradictions in results may be attributed to the fact that these studies have not considered the basal concentration of Se and Se status of animals before supplementation, and thus, a clear overview could not be achieved. In this study, the CON diet contained Se at 0.034 mg/kg, which seems within the low limits reported for rabbits (between 0.01 and 0.15 mg/kg DM diet; 22–23). With dietary OSe supplementation, this concentration increased to 0.334 mg/kg DM diet and was associated with significant improvements in blood serum and seminal plasma Se concentrations, physiological responses, and reproductive performance of rabbits fed the OSe diet. The increase in Se concentrations in the blood serum and seminal plasma started on week 4 and stabilized until week 12 of the experiment. This pattern of increase might have indicated that Se homeostasis was achieved at the supplemental Se concentration used in this study. The response curve of Se is *U*-shaped, with a constringed window between deficient and excessive intakes. Either a deficient level or an excessive level has been shown to affect the redox balance, leading to drastic adverse health consequences (7). Accordingly, the positive effects observed in the present study due to dietary Se supplementation on the physiological variables and reproductive performance of male rabbits support the relevance of the dietary Se concentration used in this study. However, it is still difficult to confirm whether such enhancements in health status and fertility of rabbit bucks were due to the fulfillment of basal Se requirements or due to the beneficial effects of supplemental OSe.

Rectal temperature is a vital indicator of the thermal environment. An increase of 1°C or less in rectal temperature is sufficient to diminish performance in a majority of livestock species, which makes this parameter a very sensitive indicator of animals' physiological response to heat stress (25). In this study, THI values (THI = 27.21–30.43) indicated that the rabbits were reared under moderate heat stress to extremely severe heat stress conditions (12). Rabbits have a thick hair coat with a low number of functional sweat glands, so the main mechanism by which rabbits can lose excess produced heat is by increasing the panting rate (respiration rate). In our study, rabbit bucks treated with OSe exhibited an ~0.5°C decrease in rectal temperature compared to the CON-treated rabbits. This reduction in rectal temperature, however, was not accompanied by an increase in respiration rate (Figure 3), indicating the positive effects of OSe on metabolism. Supplementation with OSe significantly improved the blood serum albumin levels (Figure 3). Albumin is responsible for 75 to 80% of the vascular colloidal osmotic pressure, in addition to its ability to maintain blood plasma expansion during heat acclimatization (3).

In addition, OSe treatment improved the blood serum glucose concentration. Adequate glucose concentration is pivotal for energy availability, liver glycogen synthesis, and normal gene expression of liver phosphoenolpyruvatecarboxy-kinase (26). These physiological events might contribute to energy production by animals and might provide energy for cellular metabolism without additional heat production (3). The other mechanism that could be involved in alleviating the negative effects of heat stress on rabbits fed the OSe diet is through improving ROS scavenging activity. Se is a structural element of numerous functional selenoproteins, including GSH-Px, iodothyroninedeiodinases, and thioredoxin reductases (5, 6). This role of Se was indicated in our study because the OSe-supplemented rabbits had higher blood serum GSH-Px activity than those fed the CON diet (Figure 3).

Rabbit bucks kept under heat stress conditions exhibit suppression of testosterone secretion, libido, and spermatogenesis. In the present study, inclusion of OSe in the diet of rabbit bucks reduced the reaction time (interval from introduction of a doe to ejaculation) by ~54%. The libido is controlled by many factors, such as the nervous system, hormones, and sexual pheromones, among which the hypothalamus–pituitary–testis axis is very important (27). Accordingly, this improvement in the libido of the OSe-treated rabbits could be attributed to the improvement in both testosterone concentration (28) and physiological adaptation (lower rectal temperature) with heat stress (3).

In this study, an obvious improvement in testicular architecture (Figure 7) was observed in the rabbits fed the OSe diet, as seminiferous tubules of rabbits fed the OSe diet were crowded by spermatid and sperm cells, which was associated with the high sperm concentration. These enhancements could be mainly attributed to the antioxidant activity of Se. Se is involved in the synthesis of a specific type of protein known as selenoproteins, which have been detected in the testis (6). Most selenoproteins, such as GSH-Px, act as carriers of Se from blood to different organs, including the testis, which diminishes the generation of unnecessary free radicals in the testis (6). In this study, the concentration of GSH-Px increased significantly in rabbits fed the OSe diet (Figure 3), providing protection to both male gametes and Leydig cells from destruction induced by oxidative stress, thus improving the sperm and testosterone concentrations.

Supplementation with OSe improved the semen ejaculate volume (Figure 4) and seminal plasma composition (protein profile, fructose concentration, and antioxidant activity, Figure 5), indicating certain improvements in accessory sex gland functions. Oldereid et al. (29) reported that the male accessory sex glands contain considerable concentrations of Se (1.3 and 1.5 μg/g dry weight in prostate and seminal vesicles, respectively), indicating the importance of Se for the functions of these glands. Hawkes et al. (30) reported that Se supplementation can positively change the composition of the accessory sex gland (prostate gland and seminal vesicles) secretions. Overall, the positive effects of Se on accessory sex gland functions could be mediated directly by Se antioxidant activity or indirectly by increased testosterone concentration, as adequate testosterone concentration is pivotal for maintaining male accessory gland functions, such as protein and fructose synthesis (31). In rabbits, positive associations have been reported between albumin and sperm motility and the percentage of normal sperm (32). Such associations are probably associated with the antioxidant activity of albumin, which provides membrane protection. In addition to membrane protection, albumin modulates cholesterol efflux from the sperm membrane, playing a role in sperm capacitation and the acrosome reaction (32). Additionally, fructose is an essential nutrient for spermatozoa and is metabolized and converted to pyruvate and lactate, which supports sperm motility (33). Supplementation with OSe improved sperm cell membrane integrity, as indicated by the high percentages of integrated sperm membranes and intact acrosomes and low seminal plasma ALT and MDA concentrations. This effect could be attributed to the Se antioxidant activity, which compromises the negative effects of free radicals on lipid peroxidation of the sperm plasma membrane (9). Considering the seminal plasma protein profile, in our study, four protein bands (Table 2, Figure 6), at 19, 21.5, 30, and 44 kDa, were detected in only the seminal plasma of rabbits fed the OSe diet. Casares-Crespo et al. (34) isolated a 19 kDa protein band, corresponding to the lipocalin allergen Ory c 4 precursor, from seminal plasma of rabbits, which was suggested to be related to improved fertility. Additionally, Taha et al. (35) found a positive association between the seminal plasma level of the 21.5 kDa protein band and sperm membrane integrity and intact acrosomes. Furthermore, the 30 kDa protein band is closely related to a family of proteins called binder sperm proteins (BSPs) in the seminal plasma of many mammals, including rabbits, rats, bulls, goats, and rams (36, 37). These proteins play many pivotal roles in male fertility, such as in (1) fertilization and early embryonic development *in vitro* (36), (2) the interaction between sperm and the oviduct epithelium of cows (37), and (3) increasing the number of heparin-binding sites on the sperm surface, controlling sperm capacitation (38). Souza et al. (39) reported that the 44 kDa protein in ram seminal plasma is phosphoglycerate kinase, which catalyzes the first ATP generation step in the central metabolic pathway and is important for sperm motility.

In our study, the level of a 35 kDa protein band was ~11-fold higher in the seminal plasma of rabbits fed the CON diet than in that of rabbits fed the OSe diet (Table 2, Figure 6). In rabbits, this protein is a major structural protein in the sperm tail fibrous sheath (40). Therefore, the presence of this protein in the seminal plasma of rabbits fed the CON diet indicates the destabilization of sperm membrane structures (lower sperm cell membrane integrity and intact acrosome levels).

With regard to the fertility of rabbit bucks, the reproductive performance of females mated with males fed the OSe diet was higher than those mated with males fed the CON diet (Table 3); however, it was still lower than that reported in other studies for this breed (V-line) under standard environmental conditions (28, 41). In tropical and sub-tropical conditions, breeding of rabbits is completely blocked in most rabbit farms due to low reproductive performance and associated health problems (1, 3). Although, dietary OSe supplementation did not completely improve reproductive performance of females to be comparable to that obtained during standard environmental conditions, it could be applied as an alternative strategy to extend the breeding season of rabbits during heat stress periods.

The improvement of reproductive performance of females mated with males fed the OSe diet seems to be an expected result, as all the fertility-related variables were better in the males fed the OSe diet than in those fed the CON diet. Previous studies have supported the positive relationship between sperm motility, sperm morphology, sperm concentration, and male fertility (19–28). Additionally, Se might have a role in embryo survival; in this context, knockout of genes encoding various selenoproteins in mice, such as GPx5 and GPx4, was related to deaths during early embryogenesis, increased abortion rates, and embryonic developmental defects (5).

## Conclusion

In this study, under heat stress conditions, supplementing the basal diet of rabbit bucks containing 0.034 mg Se/kg DM with 0.3 mg OSe/kg DM diet could improve the heat tolerance and health status of rabbit bucks, leading to substantial improvements in semen quality traits and subsequent fertility. Further studies are required to show the standard concentration of basal dietary Se required by rabbit bucks and the benefits of supplemental dietary Se on fertility and health status under different environmental conditions using an adequate number of animal experimental units.

## Data Availability Statement

All of the data produced and analyzed in the present study are included in the article as tables. The corresponding author will respond to requests concerning the raw data, and reasonable accommodations will be provided.

## Ethics Statement

The animal study was reviewed and approved by Department of Animal and Fish Production, Faculty of Agriculture, Alexandria University, Egypt.

## Author Contributions

All authors listed have made a substantial, direct and intellectual contribution to the work, and approved it for publication.

## Conflict of Interest

The authors declare that the research was conducted in the absence of any commercial or financial relationships that could be construed as a potential conflict of interest.
